# Effect of feeding frequency of a diet containing soya hulls on the food intake and behaviour of dogs*

**DOI:** 10.1017/jns.2014.34

**Published:** 2014-12-08

**Authors:** Tabyta T. Sabchuk, Juliana R. Silva, Francielle O. Marx, Ananda P. Felix, Alex Maiorka, Simone G. Oliveira

**Affiliations:** Universidade Federal do Paraná, Rua dos Funcionários, 1540, 80035-050, Curitiba, PR, Brazil

**Keywords:** Canine nutrition, Fibre sources, Obesity, BW, body weight, CF, crude fibre, CP, crude protein, IF, insoluble fibre, ME, metabolisable energy, MER, metabolisable energy requirement, SF, soluble fibre, SH, soya hulls

## Abstract

Dietary fibre may promote satiety and reduce energy consumption per gram of food. Associated with feeding management, dietary fibre may contribute to reduce anxiety in dogs submitted to food restriction to lose weight. The objective of the present study was to evaluate the food intake and the behaviour of dogs fed diets without soya hulls (0SH) or with soya hulls (16SH) once (1×) or twice (2×) daily. Eight adult Beagle dogs, with 11·3 (se 1·6) kg body weight (BW), 4·1 (se 0·1) years of age and body condition score between 4 and 7, were distributed in 4 × 4 Latin square design (*n* 8/treatment, 5 d/period) in a 2 × 2 factorial arrangement (0SH *v.* 16SH; 1× *v.* 2×). Food allowance was 50 % in excess of the daily metabolisable energy (ME) requirements; food residues were weighed. The behavioural test consisted in continuously observations for 24 h, using the scanning sampling technique (observations every 10 min). General behaviours, such as sleeping, barking, standing and others were recorded. Food intake in g/kg BW^0·75^ per d of 0SH and 16SH was not different (*P* > 0·05). However, dogs fed the 0SH diet presented higher (*P* > 0·05) energy intake (kJ/kg BW^0·75^ per d; *P* < 0·05) compared with those fed the 16SH diet. Dogs fed 2× daily had higher intake, both in g and in kJ, compared with those fed 1 × , independently of diet. There was no interaction between diets and feeding frequency (*P* > 0·05). No behavioural differences were observed (*P* > 0·05). The diet containing 16SH, despite reducing ME intake, did not restrict food intake (in g). Dogs fed 1× per d had lower food intake, possibly due to gastric capacity limitation.

Pet obesity has become increasingly common in many countries^(^^1^^)^. In the USA, it is estimated that approximately 40 % of the dogs are overweight or obese^(^^2^^)^. Therefore, different feeding management strategies have been applied together with different diets to control and/or to reduce body weight (BW). The inclusion of fibre in foods may help, as they dilute dietary energy and may provide the sensation of satiety in the short and long terms^(^^3^^)^.

Bosch *et al.*^(^^4^^)^ evaluated the influence of dietary macronutrients on general dog behaviour and reported that diets with high-fibre inclusion may promote satiety, as demonstrated by the increased inactivity and lower level of arousal compared with those fed low-fibre diets. According to Asakawa *et al.*^(^^5^^)^, the sensation of hunger in rats increase anxiety. In dogs, this may contribute to the expression of behavioural problems related with anxiety^(^^3^^)^.

Both food allowance and fibre physical–chemical characteristics, including fermentability, solubility and viscosity, may determine satiety in animals^(^^2^^,^^3^^)^. Several studies indicate that fibre effects on satiety may be mainly attributed to soluble fibres (SF)^(^^6^^)^, which are more viscous than the insoluble fibres (IF), reducing the food passage rate and stimulating the production and secretion of hormones related to satiety in the gastrointestinal tract^(^^2^^)^. However, the dietary inclusion of SF is limited, as they may increase faecal moisture^(^^7^^)^.

Soya hulls (SH), the fibre source used in the present study, contain mainly IF (IF-to-SF ratio of between 15·4:1 and 5·0:1^(^^8^^)^). It is widely available in the market and may reduce costs for the pet food industry. Therefore, the objective of the present study was to evaluate food intake, metabolisable energy (ME) intake and the behaviour of dogs fed diets containing or not SH once or twice daily.

## Materials and methods

The experiment was approved by the Committee of Ethics on Animal Use of the sector of Agrarian Sciences of the Federal University of Paraná, Curitiba, PR, Brazil, under protocol no. 019/2012.

### Animals and facilities

Eight adult Beagle dogs (four males and four females), with 11·3 (se 1·6) kg average BW and 4·1 (se 0·1) years of age and body condition score between 4 and 7 were studied. All dogs were previously submitted to clinical and physical examinations, vaccinated and de-wormed. Dogs were individually housed in concrete kennels with a solarium (5 m long × 2 m wide).

### Diets

The analysed composition of the SH included in the diet was: 13·0 % crude protein, 72·0 % total dietary fibre, 65·5 % IF, and 6·5 % SF at an IF:SF ratio of 9·97:1. SH were included in the diet replacing maize. Two diets based on maize and poultry by-product meal were formulated. The diet with no inclusion of soya hulls (0SH) contained 28·0 % crude protein, 93·4 % DM, 14·8 % diethyl ether extract in acid hydrolysis, 14·4 % total dietary fibre, 0·2 % SF, 14·1 % IF; and 17·9 MJ/g ME. The diet with 16 % soya hulls (16SH) contained 29·3 % crude protein, 93·4 % DM, 12·8 % diethyl ether extract, 24·9 % total dietary fibre, 6·2 % SF, 18·7 % IF and 15·7 MJ/g ME. The diets were ground in a mill using 1·0 mm mesh, and were extruded in a single-screw extruder (E-130; Ferraz). Dietary DM, crude protein and diethyl ether extract contents were analysed according to the Association of the Official Analytical Chemists^(^^9^^)^. The contents of total dietary fibre, SF and IF were determined according to the method of Prosky *et al.*^(^^10^^)^. Gross energy was determined in a bomb calorimeter (Parr Instrument Co. model 1261). The ME was determined *in vivo* in a previous total faecal collection digestibility trial with eight adult Beagle dogs, totalling eight replicates per treatment (unpublished results; Tabyta T. Sabchuk, 2013), according to the Association of American Feed Control Officials^(^^11^^)^.

### Food intake and behavioural evaluations

The two diets (0SH or 16SH) were offered by two feeding managements, fed once, at 08·00 h, or twice, at 08·00 and 16·00 h, daily. The allowance of the 0SH diet was 50 % in excess of the National Research Council(1) recommendations of ME maintenance requirements (MJ/d) of dogs, according to the equation: 0·54 × BW^0·75^ (kg) + 50 %. This allowance was adopted based on the observed feed residues. The 16SH diet was fed at the same amount (in g of DM) as the 0SH diet. Food intake (offer-residues) was calculated for each meal. The diets were fed for 5 d. Although this was not a digestibility trial, the period of 5 d was used because, according to the Association of American Feed Control Officials^(^^11^^)^, this is the period required for dog's adaptation to the diets and facilities. Also, fibres promote satiety particularly in the short term due to their filling effect in the gastrointestinal system during food intake^(^^2^^,^^12^^).^

Dog behaviour was evaluated always on the 4th day of the experimental period for 24 h using the scanning sampling technique^(^^13^^)^. Behaviours were recorded every 10 min, and are expressed as frequency (%) of occurrence. The following behaviours were recorded: idle standing (on 4 ft), idle sitting (leaning on the stretched front legs and flexed hind legs), resting (lying on ventral or latero-lateral position and eyes closed), drinking, eating, alert (standing and attentive to movements), socialising (interaction with dogs of the neighbouring kennels), stereotypies (uninterrupted abnormal behaviour)^(^^14^^)^, scratching, walking and self-grooming.

### Statistical analysis

Data were analysed according to a replicated Latin square design (four treatments × four periods) in a 2 × 2 factorial arrangement (SH dietary inclusion level and feeding management). Every 2 dogs were fed one of the diets in each period, totalling eight replicates per treatment. The experimental unit was one dog. The sum of the squares of the ANOVA was separated in effects of animal, period, SH inclusion level and feeding management effects, as well as effects of the interaction between SH inclusion level and feeding management. In the *F* test, differences with *P* < 0·05 were considered significant.

The frequencies (%) of the observed behaviours were analysed by the test of Kruskal–Wallis, with *P* < 0·05 indicating significant difference. All analyses were carried out using SAS statistical package (Statistical Analysis System, version 8.2; SAS Inst. Inc., Cary, NC).

## Results

Food intake was not different between diets 0SH and 16SH (*P* > 0·05; Table 1). However, the dogs that consumed the 0SH diet presented greater ME intake (*P* > 0·05; Table 1) compared with those fed the 16SH diet. In addition, intake, both in grams and kJ, was greater in dogs fed twice daily compared with those fed once daily. There was no interaction between dietary SH inclusion and feeding frequency (*P* > 0·05). No differences were observed in dog's behaviour (Table 2).

**Table 1.** Food intake in g, food intake per body weight (BW) and metabolisable energy (ME) intake of eight dogs fed diets without soya hulls (0SH) or with soya hulls (16SH) once (1×) or twice (2×) daily

Factors	Treatments	Intake (g)*	Intake (BW^0·75^)†	ME intake‡
Diets (D)	0SH	297·31	47·06	843·30
	16SH	293·34	46·43	732·79
Feeding frequency (FF)	1 ×	258·02	40·74	689·02
	2 ×	332·64	52·75	887·07
sem		12·452	1·836	31·832
*P* value				
D		0·854	0·836	0·037
FF		0·016	<0·001	<0·001
D × FF		0·182	0·195	0·285

*Food intake in g (g ‘as is’/dog per d).

†Food intake per BW^0·75^ (g/kg BW^0·75^ per d).

‡ME intake (kJ ME/kg BW^0·75^ per d).

**Table 2.** Median frequency of behaviours observed in eight dogs fed diets without soya hulls (0SH) or with soya hulls (16SH) once (1×) or twice (2×) daily

Behaviour	Treatments	Median (%)	*P* value*
Idle lying	0SH 1 ×	8·62	0·523
	16SH 1 ×	6·21	
	0SH 2 ×	8·62	
	16SH 2 ×	8·97	
Idle sitting	0SH 1 ×	6·90	0·951
	16SH 1 ×	6·21	
	0SH 2 ×	7·90	
	16SH 2 ×	7·59	
Idle standing	0SH 1 ×	2·76	0·342
	16SH 1 ×	4·48	
	0SH 2 ×	5·52	
	16SH 2 ×	3·79	
Sleeping	0SH 1 ×	62·07	0·759
	16SH 1 ×	64·83	
	0SH 2 ×	58·62	
	16SH 2 ×	62·41	
Self-grooming	0SH 1 ×	3·79	0·758
	16SH 1 ×	2·41	
	0SH 2 ×	2·88	
	16SH 2 ×	2·76	
Walking in the pen	0SH 1 ×	2·41	0·573
	16SH 1 ×	2·76	
	0SH 2 ×	1·38	
	16SH 2 ×	2·41	
Other†	0SH 1 ×	10·69	0·953
	16SH 1 ×	11·03	
	0SH 2 ×	12·07	
	16SH 2 ×	10·69	

*Probability (*P*) test of Kruskal–Wallis.

†Other is relative to the other observed behaviours, which frequency was lower than 2 % of the time of observation.

## Discussion

Considering the increasing number of obese dogs, foods to control BW need to be developed. These foods may contain restricted energy levels, which may cause the dogs to feel hungry, increasing their anxiety and leading to undesirable behaviours. Therefore, food formulas for obese dogs should maintain satiety for long periods and, at the same time, allow for the healthy control of BW.

Hunger is controlled by the presence of food in the gastrointestinal tract and by blood levels of nutrients. It is difficult to identify and to measure hunger, the easiest way to assess hunger is by evaluating satiety^(^^15^^)^. However, there is no definite protocol to measure satiety^(^^15^^)^ in terms of hunger inhibition and that could be measured by the time interval until the next meal or by the amount of food intake in the next meal^(^^16^^)^.

Some studies have evaluated the effect of fibrous diets on the satiety of dogs^(^^12^^,^^17^^)^. According to Burton-Freeman^(^^16^^)^, dietary fibre influences satiety due to its physical and chemical characteristics, such as volume, solubility and viscosity, in addition to reducing dietary energy density. Some studies indicated that soluble and fermentable fibres exert stronger effects on satiety than IF^(^^2^^,^^6^^,^^18^^)^. SF delay gastric emptying, making the digesta remain longer in the gastrointestinal tract^(^^19^^)^, in addition to possibly influencing the production of hormones related to satiety^(^^1^^)^. Cummings *et al.*^(^^20^^)^ found that fermentable fibres affected blood ghrelin levels in human subjects. On the other hand, Bosch *et al.*^(^^2^^)^, evaluating the supply of a diet-containing low-fermentation fibre (8·5 % cellulose) or of a diet with high-fermentation fibre (combination of inulin and beet pulp, at 8·5 % inclusion level) to dogs, did not observe any difference in ghrelin concentration or in food intake.

In a previous study, carried out in our laboratory (unpublished results, Tabyta T. Sabchuk, 2013), the dietary inclusion of SH did not affect diet palatability, which suggests that food intake in the present experiment was not influenced by taste. Although food intake was not different, dogs fed the diet with 16SHhad lower ME intake than those fed the diet with 0SH. This demonstrates that SH fibre reduces dietary energy, and hence could be used as a source of fibre for energy dilution in dog foods. Consistent results were obtained by Jewell *et al.*^(^^21^^)^, who evaluated a low-fibre control diet (2 % crude fibre) and a high-fibre commercial diet (20 % crude fibre, but did not report the type of fibre) and also found reduced energy intake, but not food intake (g/d).

In the present study, satiety was estimated by the food intake of dogs fed once or twice daily. Despite receiving 50 % in excess of their ME requirements, they did not differ in satiety, because the intake (in grams) of the diets with or without SH was not different. However, the dogs fed once daily presented lower intake relative to those fed twice daily, independently of the diet. This may be explained by the physical theory of intake regulation. This seems to be the most effective theory to explain food intake in dogs, because in the present experiment, dogs did not stop eating when their ME intake was supplied; only when they were no longer able to eat due to the physical limitation of the gastrointestinal tract.

This may be attributed to the feeding behaviour of dog ancestors. Wild dog packs ingest large amounts of food in a short time due to larger volume and distension capacity of their stomachs relative to domestic dogs and to the competitiveness among members of the pack^(^^22^^)^.

According to Bosch *et al.*^(^^4^^)^, nutrients, like fibre, influence animal behaviour, because promotes satiety, it may reduce the frequency of motivation and feeding behaviours elicited by the anxiety caused by hunger^(^^3^^)^. According to those authors, dogs felt less hungry when fed diets containing fermentable fibres than when fed less fermentable fibres. This is consistent with the results of the present study, in which the diet did not influence dog behaviour, possibly because SH were used as fibre source, which is characterised as insoluble^(^^8^^)^ and with intermediate fermentability^(^^23^^)^.

As shown in the present study, adequate feeding management is as important to reduce/control BW as a energy-restricted diet.

Moreover, the lack of influence of feeding management on dog behaviour observed in the present study may be due to the supply of food in excess of the energy requirements of dogs submitted to both practices. This may have masked the effect of satiety when dogs were fed twice daily, independently of the diet.

It is difficult to study satiety in dogs, as it is influenced by several extrinsic and intrinsic factors, as well as their interactions and to date, there is no defined protocol for its evaluation in dogs^(^^22^^)^. It is possible that the behaviour of the dogs in this experiment was not affected by the presence of the researchers, as these evaluated the dogs from far (except at the time of food offer), did not interact with dogs during the behavioural evaluation, the dogs were habituated to the researchers, and the methodology was previously applied^(^^24^^)^. However, the use of video cameras to monitor behaviour might be better to avoid any possible influence of the presence of human subjects on dog behaviour. This might have been a limitation of the present study.

## Conclusions

The inclusion of fibre, by means of SH, in the diet does not reduce food intake (g/d) of Beagle dogs, but reduces their energy intake. Therefore, the inclusion of 16SH does not induce satiety in dogs fed food amounts that exceed their energy requirements. However, the physical capacity of their gastrointestinal tract limits food intake (in g/d) of dogs.
